# Genetic diversity analysis of a flax (*Linum usitatissimum* L.) global collection

**DOI:** 10.1186/s12864-020-06922-2

**Published:** 2020-08-14

**Authors:** Ahasanul Hoque, Jason D. Fiedler, Mukhlesur Rahman

**Affiliations:** 1 Department of Plant Sciences, North Dakota State University, Fargo, ND USA; 2 Cereal Crops Research, Edward T. Schafer Agricultural Research Center, USDA-ARS, Fargo, ND USA

**Keywords:** Flax, SNP, GBS, Genetic diversity, Linkage disequilibrium, Germplasm collection

## Abstract

**Background:**

A sustainable breeding program requires a minimum level of germplasm diversity to provide varied options for the selection of new breeding lines. To maximize genetic gain of the North Dakota State University (NDSU) flax breeding program, we aimed to increase the genetic diversity of its parental stocks by incorporating diverse genotypes. For this purpose, we analyzed the genetic diversity, linkage disequilibrium, and population sub-structure of 350 globally-distributed flax genotypes with 6200 SNP markers.

**Results:**

All the genotypes tested clustered into seven sub-populations (P1 to P7) based on the admixture model and the output of neighbor-joining (NJ) tree analysis and principal coordinate analysis were in line with that of structure analysis. The largest sub-population separation arose from a cluster of NDSU/American genotypes with Turkish and Asian genotypes. All sub-populations showed moderate genetic diversity (average *H* = 0.22 and *I* = 0.34). The pairwise *F*_*st*_ comparison revealed a great degree of divergence (*F*_*st*_ > 0.25) between most of the combinations. A whole collection mantel test showed significant positive correlation (r = 0.30 and *p* < 0.01) between genetic and geographic distances, whereas it was non-significant for all sub-populations except P4 and P5 (r = 0.251, 0.349 respectively and *p* < 0.05). In the entire collection, the mean linkage disequilibrium was 0.03 and it decayed to its half maximum within < 21 kb distance.

**Conclusions:**

To maximize genetic gain, hybridization between NDSU stock (P5) and Asian individuals (P6) are potentially the best option as genetic differentiation between them is highest (*F*_*st*_ > 0.50). In contrast, low genetic differentiation between P5 and P2 may enhance the accumulation of favorable alleles for oil and fiber upon crossing to develop dual purpose varieties. As each sub-population consists of many genotypes, a Neighbor-Joining tree and kinship matrix assist to identify distantly related genotypes. These results also inform genotyping decisions for future association mapping studies to ensure the identification of a sufficient number of molecular markers to tag all linkage blocks.

## Background

Flax (*Linum usitatissimum* L.) is an ancient crop, grown throughout the world to be sold at market. Domestication events have separated flax into two broad types: seed flax and fiber flax [1]. Seed flax is mainly grown for oil which is rich in omega-3 fatty acid. Preference of flaxseed in human diet is increasing rapidly due to its high dietary fiber, omega-3 oils, and anti-carcinogenic lignans [2]. Flaxseed oil is also used in paints and varnishes for its unique drying properties [3]. On the other hand, fiber flax is grown to harvest fiber for making linen cloth. In recent years, high value product development has been the prime target of fiber industry from flax stem [4].

Diversity is an important characteristic of a sustainable breeding program. More diversity of germplasm provides the breeder better options for selecting parents to develop need-based cultivars. Diversity is also important for association mapping as the broadest diversity is correlated with a rapid LD decay [5]. Diversity in genetic materials occurs due to variation in phenotypic appearance and genotypic background. Initially, the diversity of flax germplasm was assessed based on morphological parameters [6, 7], and biochemical marker such as isozymes [8, 9]. However, morphometric diversity often leads to false prediction as morphological characteristics are plant developmental stage dependent and environment sensitive [10]. Morphological characterization is also labor intensive and time consuming. In addition, isozyme markers are affected by plant developmental stage [11, 12] and are available for only a limited number of loci [13, 14]. The limitations of morphological and biochemical markers has led to the development of DNA based markers which are environment independent and do not require previous pedigree information [15]. Molecular marker-based diversity is more precise and economic as it allows breeders to select unrelated individuals among thousands of genotypes within a short period of time which in turn reduces field workload by evaluating only unrelated genotypes. Different molecular marker techniques such as RAPD, AFLP, ISSR, SSR and IRAP has been used to assess the genetic diversity of flax germplasm [16–22]. The availability of a flax reference genome [23] has created the opportunity of discovery and exploitation of SNP markers, which are abundant and well distributed throughout the genome.

North Dakota State University runs a moderate size flax breeding program to develop improved cultivars with conventional breeding methods. The program is being hampered by the narrow genetic base of parental stocks, as the same sets of parents have been crossed repeatedly in different combinations. To enrich the parental stock, the program now desires to incorporate diverse germplasm to existing parental stock. To speed up the selection procedure and increase the genetic gain per year, the program also desires to apply marker-assisted and genomic selection techniques by exploring marker-trait association through genome-wide association mapping. To identify marker trait association, quantitative trait loci (QTL) and association mapping (AM) approaches are commonly used. QTL mapping is done by tracking the cosegregation of QTL and marker loci in biparental mapping populations and reveals low resolution regions due to the relatively low recombination rates of a single cross. AM reveals marker trait association by utilizing linkage disequilibrium (LD) of germplasm collections [24]. Although AM yields high resolution loci by exploiting historical recombinant events, it is affected by population structure which results in false positive association. Thus null or weak population structure and a low level of relatedness among individuals of the germplasm collection is desirable which leads to rapid LD decay and increases the power of marker detection [5].

In this study, we genotyped 350 flax germplasm accessions using 6200 informative SNP markers. The objectives were (1) to explore genetic diversity and differentiation among the genotypes, (2) to investigate the potential of the collection as parental resource and (3) to assess the suitability of the collection for marker-assisted breeding.

## Results

### SNP profile

The selected 6200 SNPs were distributed across 15 chromosomes with an average marker density of 1 per 51.17 kb Chromosome Lu1 and Lu4 contained highest (550 SNPs, 8.88%) and lowest (299 SNPs, 4.82%) number of SNPs, respectively. The SNP density was lowest on chromosome Lu4 (66.34 kb) and was highest on chromosome Lu13 (36.95 kb) (Table 1, Figure S3). The occurrence of transition SNPs (3532 SNPs) was more than that of transversions (2668 SNPs) with a ratio of 1.32. The frequency of C/T transitions was highest (28.61%) and C/G transversions were lowest (9.56%). Both A/G and C/T transitions occurred in similar frequencies (i.e. A/G 28.35% and C/T 28.61%), whereas the frequencies of four transversions were: A/C 11.61%, A/T 10.40%, C/G 9.56%, G/T 11.45% (Table 2). The inbreeding coefficient within individuals (*F*_*is*_), fixation index (*F*) and observed heterozygosity (*Ho*) of all the markers were 1, 1 and 0 respectively as all were homozygous. The Shannon’s information index (*I*) of all markers ranged from 0.03 to 0.70 with a mean value of 0.34.

**Table 1:** Distribution of SNPs

Chromosome	No. of SNPs	% SNPs	Start position ^a^	End position ^a^	Length (Mb)	Density (Kb)
Lu1	550	8.87	48,002	28,940,544	28.89	52.53
Lu2	402	6.48	343,539	25,278,102	24.93	62.03
Lu3	485	7.82	56,610	26,551,417	26.49	54.63
Lu4	299	4.82	20,788	19,857,012	19.83	66.34
Lu5	424	6.84	58,098	17,649,206	17.59	41.49
Lu6	366	5.90	29,887	17,856,972	17.82	48.71
Lu7	389	6.27	3623	18,287,460	18.28	47.00
Lu8	358	5.77	81,017	23,662,693	23.58	65.87
Lu9	412	6.65	124,789	21,763,401	21.63	52.52
Lu10	308	4.97	199,991	17,833,309	17.63	57.25
Lu11	454	7.32	76,724	19,841,794	19.76	43.54
Lu12	425	6.85	52,319	20,832,003	20.77	48.89
Lu13	552	8.90	14,015	20,413,108	20.39	36.95
Lu14	423	6.82	24,838	19,367,496	19.34	45.73
Lu15	353	5.69	38,217	15,613,904	15.57	44.12
Mean	413.33					51.17

^a^position given in bp

**Table 2:** Transition and transversion SNPs across the genome

SNP type	Model	No. of sites	Frequencies (%)	Total (percentage)
Transitions	A/G	1758	28.35	3532 (56.97%)
	C/T	1774	28.61	
Transversions	A/T	720	11.61	2668 (43.03%)
	A/C	645	10.40	
	G/T	593	9.56	
	G/C	710	11.45	

The expected heterozygosity (*He*) ranged from 0.08 to 0.53 with a mean value of 0.30. The polymorphic information content (*PIC*) ranged from 0.07 to 0.47 with a mean value of 0.24 (Table S2). Sub-population wise marker diversity parameters are presented in supplementary Table S3.

### Population structure

**Fig. 1:**
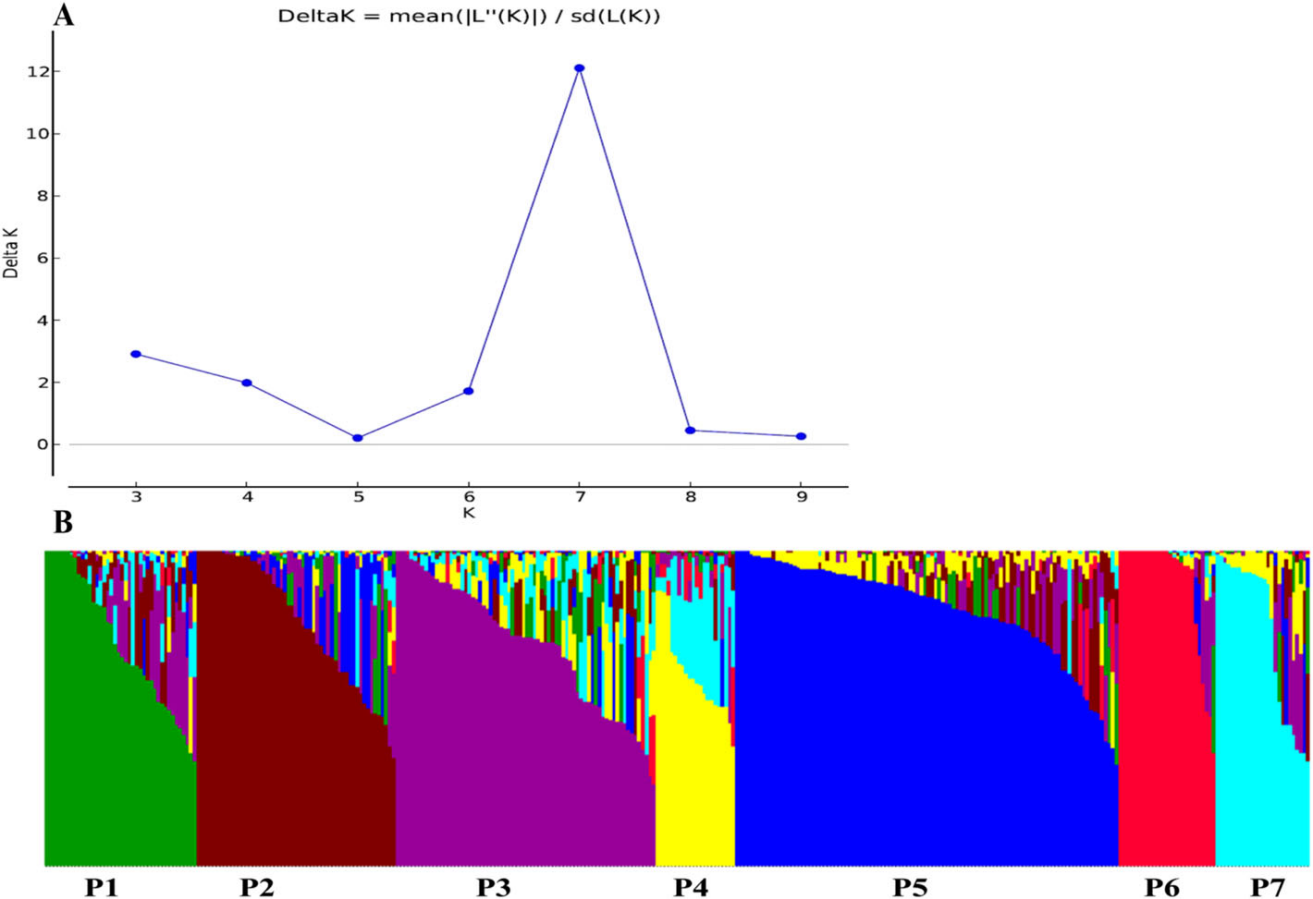
Clustering of whole collection using 6200 SNP markers. **a** Graphical representation of Delta K for different number of sub-population determined by Evanno’s method. **b** Estimated population structure of 350 flax genotypes on K = 7 according to Delta K

The whole collection was divided into seven sub-populations based on structure analysis using the Delta K approach (Fig. 1a). The NDSU released and other American genotypes were grouped under sub-population-5 (P5) whereas European (Hungary), Turkish and Asian (India & Pakistan) genotypes were under sub-population-1 (P1), sub-population-7 (P7) and sub-population-6 (P6), respectively. Sub-population-2 (P2), sub-population-3 (P3) and sub-population-4 (P4) were composed of a mixture of genotypes of different origins (Fig. 1b). All of the sub-populations consist of oil type genotypes except sub-population-2, which consists of mostly fiber type genotypes. Among oil types, spring type seed flax belong to P5, winter types belong to P1 and P7, short large seed Indian seed flax belong to P6, Mediterranean or Argentine seed flax belong to P3 and Ethiopian forage type seed flax belong to P4. Based on individual Q matrix, the proportion of pure (non-hybrid) and admixed (containing markers assigned to more than one sub-population) genotypes in each sub-population was calculated.

**Fig. 2:**
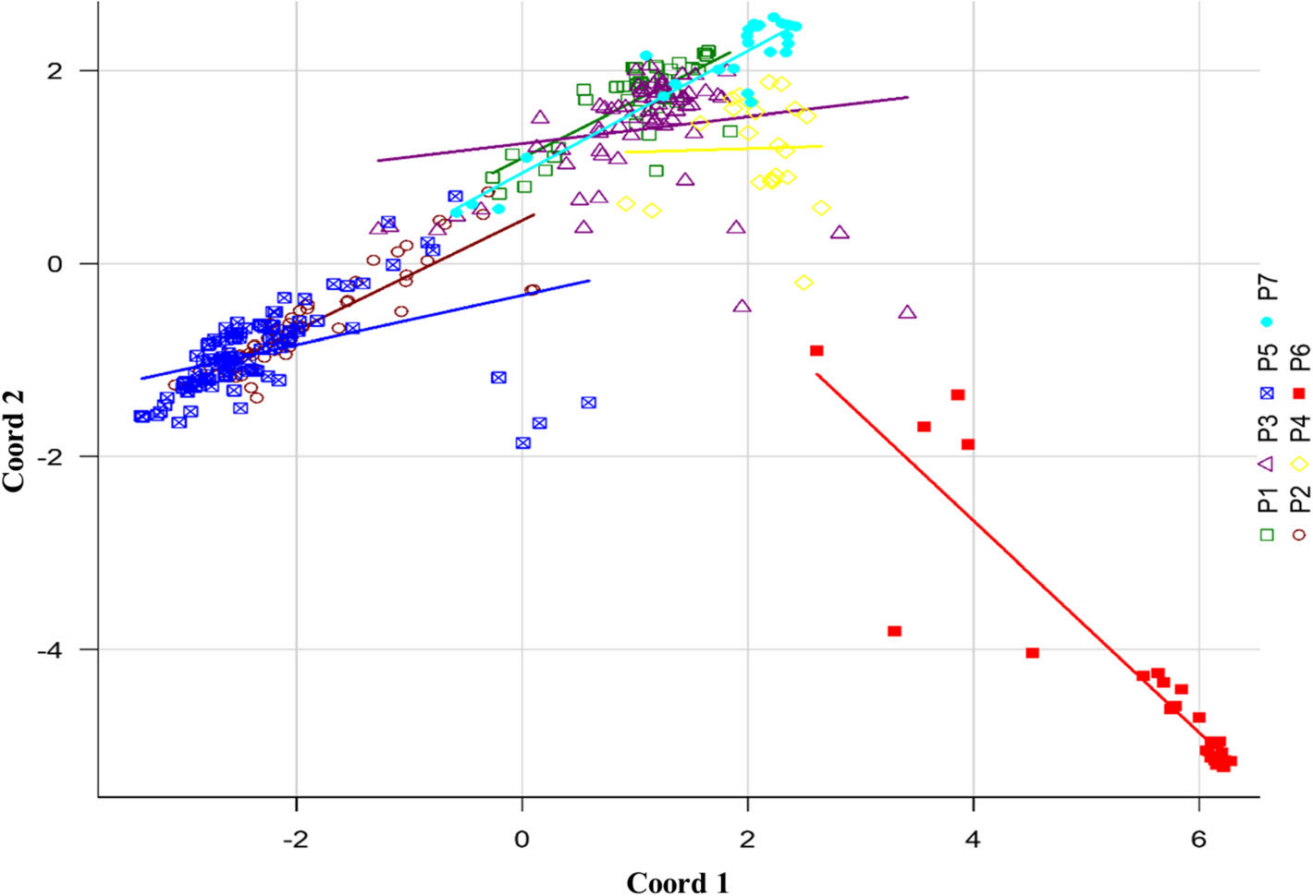
Principal coordinate analysis of SNP diversity based on genetic distance. Colors represent sub-populations identified at K = 7 in Fig. 1

**Fig. 3:**
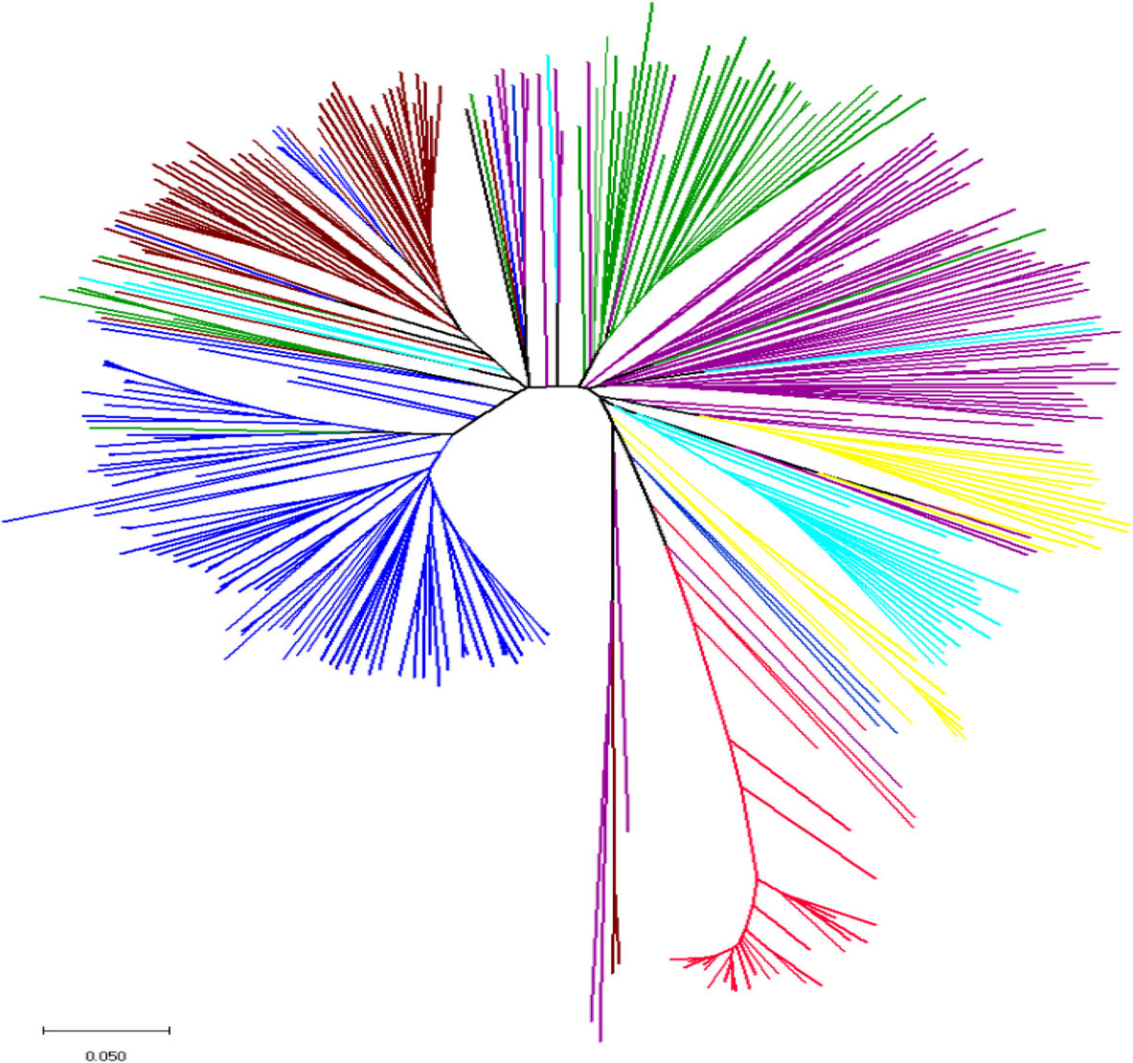
Neighbor-joining phylogenetic tree based on genetic distance matrix representing the clustering of 350 flax genotypes

The proportion of pure accessions in each sub-population ranged from 18 to 81% at a 0.7 cutoff value. The P5 and P6 contained highest percentage (81%) of pure accessions, whereas P4 contained the lowest percentage (18%) (Table 3). We also performed principal coordinate analysis (PCoA) to show the genetic similarity among sub-populations. The first two axes explained 18.49% of the total observed variation (Table S4). The PCoA revealed that NDSU released and other American genotypes (P5), Turkish (P7) and Asian (P6) genotypes were well clustered and separated from rest of the genotypes (Fig. 2). In addition to that, we also constructed phylogenetic tree based on neighbor joining (NJ) criteria (Fig. 3). The output of neighbor-joining (NJ) tree analysis was in line with that of structure analysis and PCoA.

**Table 3:** Number of pure and admixed individuals per sub-population

Sub-populations	Total no. of genotypes	0.7 cutoff		0.9 cutoff	
		No. of genotypes	% of from total	No. of genotypes	% of from total
P1	42	20	47.62	12	28.6
P2	55	35	63.64	21	38.2
P3	72	44	61.11	14	19.4
P4	22	4	18.18	0	0.0
P5	106	86	81.13	40	37.7
P6	27	22	81.48	21	77.8
P7	26	16	61.54	14	53.8
Total	350	227	64.86	122	34.9

### Population diversity

**Fig. 4:**
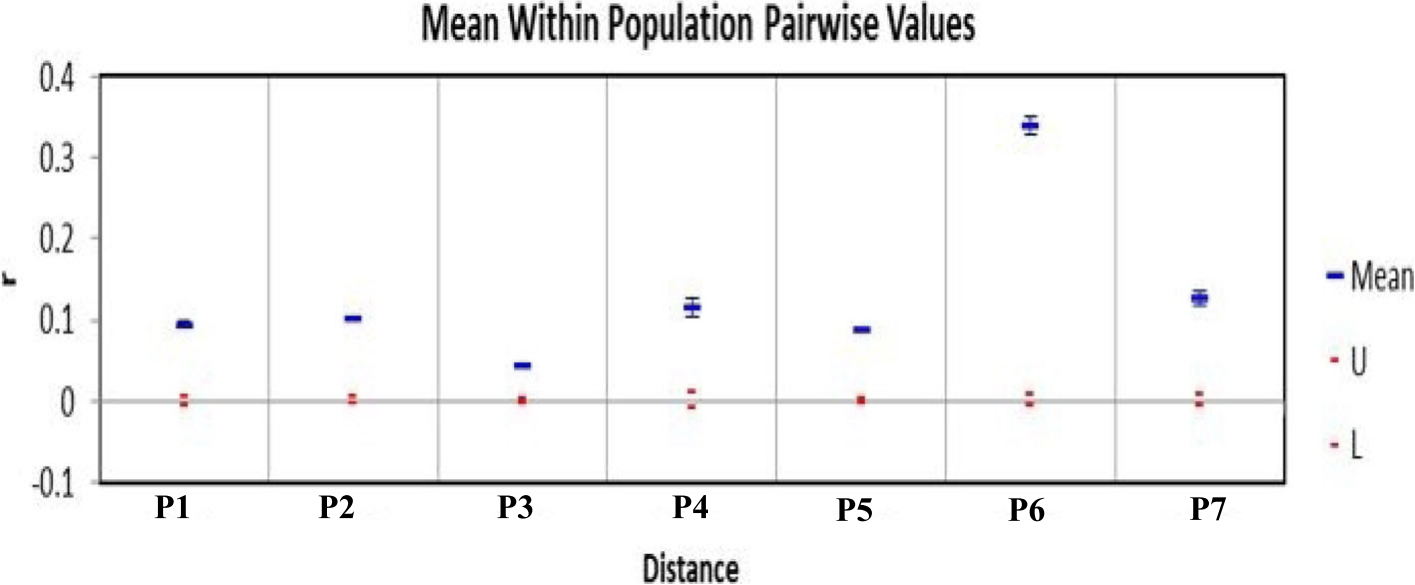
Within sub-population pairwise mean relatedness (*r*) for flax collections

In all sub-populations the percentage of polymorphic loci was greater than 60%. It was highest in P3 (97.53%) and lowest in P6 (62%). The diversity (*H*) of the seven sub-populations ranged from 0.12 (P6) to 0.28 (P3) with an average of 0.22. The Shannon’s information index (*I*) ranged from 0.21 (P6) to 0.44 (P3) with an average of 0.34. Likewise percentage of polymorphic loci and diversity, it was highest in P3 and lowest in P6. The Tajima’s D value ranged from − 0.85 (P6) to 1.50 (P3) with an average of 0.52 (Table 4). The mean pairwise relatedness (*r*) among individuals within sub-population was significant (*p* < 0.01). The P3, P5 and P1 showed lower (< 0.1) *r* values and it increased for P2 (0.10), P4 (0.11), P7 (0.12) and was highest for P6 (0.34) (Table 5, Fig. 4). The *I* and *H* were significantly and negatively correlated with relatedness (*r* = − 0.91, − 0.89 respectively and *p* < 0.01).

**Table 4:** Sub-population wise diversity parameters

Sub-populations	Polymorphic loci (%)	*Na* ^a^	*Ne* ^b^	*I* ^c^	*H* ^d^	*Uh* ^e^	Tajima’s D
P1	87	1.87	1.39	0.37	0.24	0.24	0.75
P2	87	1.87	1.33	0.33	0.21	0.21	0.40
P3	98	1.98	1.46	0.44	0.28	0.29	1.50
P4	78	1.78	1.41	0.37	0.24	0.25	0.79
P5	88	1.88	1.33	0.32	0.20	0.20	0.69
P6	62	1.62	1.18	0.21	0.12	0.13	−0.85
P7	80	1.80	1.35	0.34	0.22	0.23	0.35
Mean	83	1.83	1.35	0.34	0.22	0.22	0.52

^a^ No. of different alleles, ^b^ No. of effective alleles, ^c^ Shannon’s information index

^d^ Diversity, ^e^ Unbiased diversity

**Table 5:** Mean pairwise relatedness (r) values within sub-population

Sub-populations	P1	P2	P3	P4	P5	P6	P7
Mean	0.095	0.101	0.043	0.114	0.088	0.338	0.127
Upper mark	0.006	0.004	0.003	0.011	0.002	0.008	0.008
Lower mark	−0.005	− 0.004	−0.003	− 0.008	−0.003	− 0.007	−0.007
*P* value	0.001	0.001	0.001	0.001	0.001	0.001	0.001

### Population genetic differentiation

The AMOVA revealed that variance among sub-populations covered 28% of total variation whereas the remaining 72% of total variation accounted for variance among individuals within sub-populations (Table 6) with a *F*_*st*_ and *Nm* value of 0.28 and 0.64, respectively. All pairwise *F*_*st*_ comparisons between sub-populations were significant (*p* < 0.01).

**Table 6:** Summary of AMOVA

Sources of variation	df	SS	MS	Est. Var.	% of variation	Fixation indices	Nm
Among sub-populations	6	164,598.46	27,433.08	274.90	28	Fst: 0.28	0.64
Among individuals	343	483,200.88	1408.75	704.37	72	Fis: 1.00	
Within individuals	350	0.00	0.00	0.00	0	Fit: 1.00	
Total	699	647,799.34		979.27	100		

Most of the combinations showed a great degree of divergence (*F*_*st*_ > 0.25) [25] except few combinations such as P1 and P3 (0.13), P3 and P4 (0.13), P3 and P7 (0.13), P2 and P5 (0.16), P4 and P7 (0.17). The pairwise *F*_*st*_ > 0.50 was observed between P2 and P6, P5 and P6, P7 and P6 (Table 7). At the loci level, the genetic differentiation, *F*_*st*_ ranged from 0.01 to 0.95 with a mean of 0.29 (Table S5). We also performed kinship (IBS) analysis to facilitate the individual genotype selection for desirable cross combinations (Figure S4). The IBS coefficients ranged from 1.12 to 2. The average coancestry between any two flax genotypes was 1.41. Approximately 80% of the pairwise IBS coefficients ranged from 1.12 to 1.50 (Table S8, Figure S5).

**Table 7:** Genetic differentiation among sub-populations

	Sub-population pairwise *Fst*						
	P1	P2	P3	P4	P5	P6	P7
P1	0.00	0.00	0.00	0.00	0.00	0.00	0.00
P2	0.25	0.00	0.00	0.00	0.00	0.00	0.00
P3	0.13	0.21	0.00	0.00	0.00	0.00	0.00
P4	0.21	0.31	0.13	0.00	0.00	0.00	0.00
P5	0.27	0.16	0.21	0.32	0.00	0.00	0.00
P6	0.48	0.54	0.40	0.46	0.54	0.00	0.00
P7	0.20	0.32	0.14	0.17	0.32	0.51	0.00

Below diagonal values are pairwise *Fst* comparison. Above diagonals depicts the *P* values.

Mantel test was performed to show the correlation between geographic and genetic distance among individuals within each sub-population (Table 8).

**Table 8:** Mantel test output showing genetic and geographic distance correlation

Sub-populations	SSx ^a^	SSy ^b^	SPxy	Rxy ^c^	*P* value
P1	45,800,457.07	5,538,022,460	93,859,398.73	0.19	0.05
P2	134,861,866.3	41,553,712,025	246,361,305.5	0.10	0.13
P3	120,655,500	8,473,759.059	3,098,570.347	0.10	0.09
P4	26,416,786.96	1,683,893,955	52,933,834.97	0.25	0.01
P5	318,721,174.9	85,673,146,302	1,827,968,297	0.35	0.01
P6	92,879,654.84	3,062,447,732	179,000,333.5	0.34	0.07
P7	19,812,140.31	479,364,655.5	35,378,695.93	0.36	0.05
Whole collection	8,016,027,762.51	1,165,284,415,054.30	28,882,623,823.41	0.30	0.001

^a^ Genetic distance, ^b^ Geographic distance, ^c^ Correlation coefficient values

Individuals of P4 and P5 showed significant positive correlation between geographic and genetic distance (r = 0.251, 0.349, respectively, and *p* < 0.05) whereas it was not significant in other sub-populations (Figure S1). In the entire collection, significant positive correlation (r = 0.30 and *p* < 0.01) was revealed by mantel test.

### Linkage disequilibrium pattern

The linkage disequilibrium (LD) pattern was investigated across the entire collection, each sub-population and chromosome-wise. LD = *r*^*2*^ values decreased with the increase of distances. In all cases, mean LD was high (*r*^*2*^ > 0.80) at short distance bin (0–1 kb) and declined with increasing bin distance (Table S6). In the entire collection, the mean linked LD, mean unlinked LD and loci pair under linked LD was 0.41, 0.02 and 2.46%, respectively. The mean linked LD was highest in P6 (*r*^*2*^ = 0.50), and was lowest in P4 (*r* ^*2*^ = 0.39). In P6, highest proportion (28.22%) of total loci pair was linked, whereas it was very low (1.08%) in P3 (Table 9). We also calculated the LD decay rate. In the whole collection, LD decayed to its half maximum within < 21 kb distance. Each chromosome showed differential rate of LD decay.

**Table 9:** Linkage disequilibrium in the studied collection

Sub-populations	Mean linked LD	Mean unlinked LD	Mean LD	Loci pairs in linked LD (%)	Loci pairs in unlinked LD (%)
Whole collection	0.41	0.02	0.03	2.46	97.54
P1	0.40	0.03	0.04	3.77	96.23
P2	0.44	0.03	0.05	5.80	94.20
P3	0.48	0.02	0.02	1.08	98.92
P4	0.38	0.04	0.08	11.56	88.44
P5	0.45	0.02	0.04	4.92	95.08
P6	0.50	0.04	0.17	28.22	71.78
P7	0.39	0.04	0.06	7.04	92.96

**Fig. 5:**
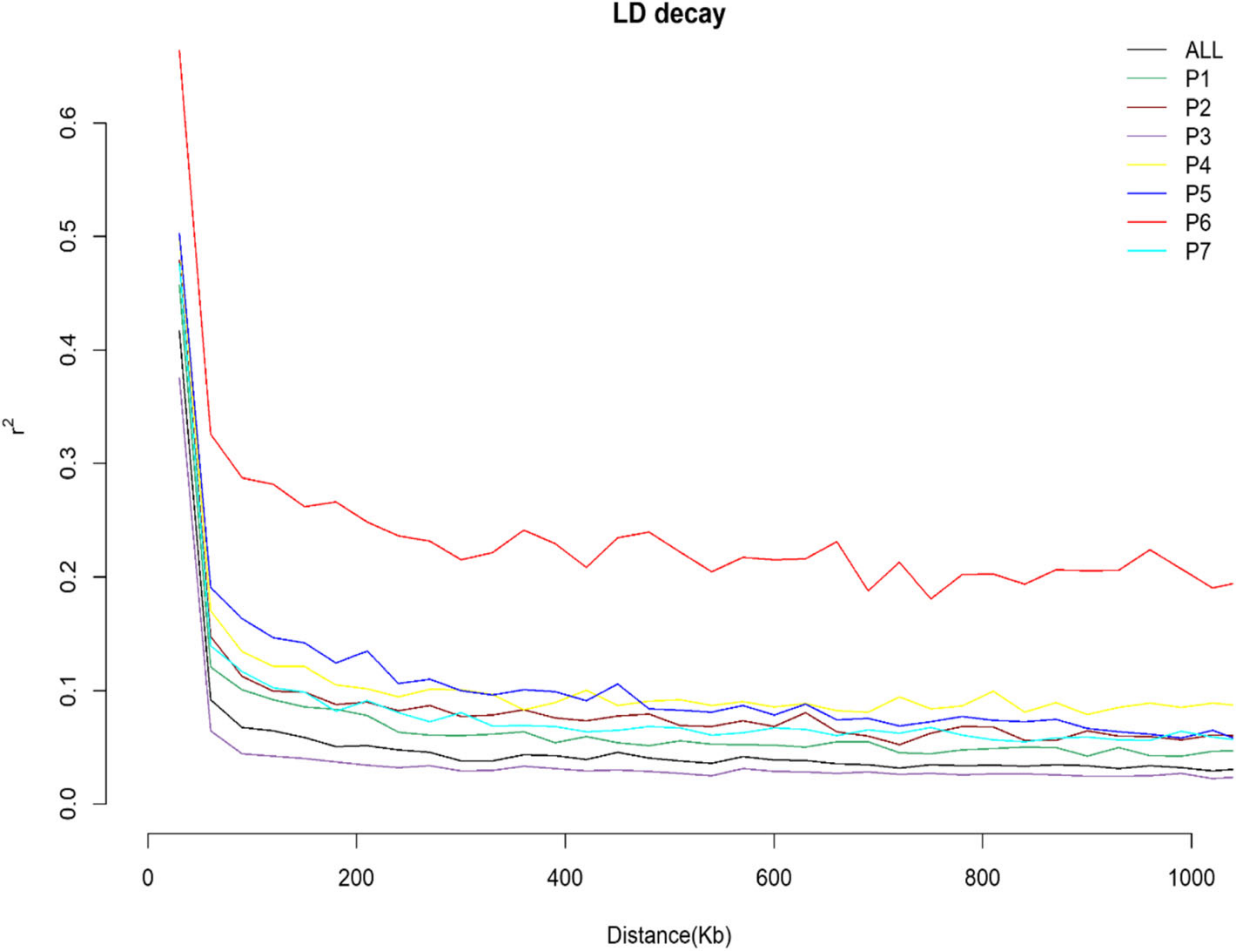
Linkage disequilibrium (LD) differences and decay pattern among sub-populations

LD persisted the longest in chromosome Lu1 (35.42 kb) and Lu3 (34.40 kb). The decay distance was shortest in chromosome Lu13 (13.71 kb) and Lu8 (14.68 kb) (Figure S2, Table S7). LD decayed to its half –maximum within < 30 kb for P1 and P3, 38.34 kb for P7, 52.68 kb for P2, < 85 kb for P4 and P5, and 1444 kb for P6 (Fig. 5, Table S7).

## Discussion

We used a total of 6200 homozygous SNP markers for diversity analysis of 350 genotypes. The used SNPs were well distributed throughout the genome. The transition SNPs were more frequent than transversion SNPs, indicating that transition mutations are more tolerable to natural selection [26]. A similar result was also found in other species such as *Camelina sativa* [27], *Camellia sinensis* [28], *Hevea brasiliensis* [29] and *Brassica napus* [30, 31], that may be due to synonymous mutations in protein-coding regions [32]. We also calculated PIC and expected heterozygosity (*He*) for each marker. The PIC determines the usefulness of any marker for linkage analysis whereas *He* determines the diversity of haploid markers [33]. We found all markers moderately or low informative as PIC value for all markers was less than 0.5 [34]. Other researchers also found similar results in flax [35], winter wheat [36, 37], rice [38] and maize [39]. Bi-allelic nature of SNP marker and probably low mutation rate [40] restrict the PIC value within 0.5. The *He* value for all markers was always greater than PIC value as PIC value become closer to *He* with more alleles and with increasing evenness of allele frequencies [33].

Selection of diversified materials is crucial for widening the genetic base of a breeding germplasm collection. In our study, based on the identified SNP markers, the different sub-populations exhibited moderate diversity (average *H* = 0.22), which is in line with our expectation as flax possesses an autogamous reproduction system. A similar level of diversity was found in one study [41], though other studies revealed both low [42, 43] and high [44, 45] level of diversity of different group of flax germplasm. The variation in results may be due to the utilization of different markers and different genotype sets by the researchers. The great homogeneity of the diversity indices of different sub-populations in the studied collection suggests that the species is durable enough to avoid the natural loss of genetic variability by drift [46]. We also calculated the Tajima’s D value to indicate the abundance or scarcity of rare alleles in different sub-population and selection mechanism behind sub-populations [47]. Sub-population P6 displayed a negative Tajima’s D value indicating presence of more rare alleles in this group or recent expansion of the group as most of the individuals of this sub-population are cultivars grown in India and Pakistan. The other six (P1 -P5 and P7) sub-populations showed positive Tajima’s D values indicating less rare alleles in those groups or recent population contraction. Previously, negative Tajima’s D values were found in flax landraces [1, 48] and fiber type flax [1] but it was positive for oil, winter and dehiscent type flax. All seven sub-populations showed significant level of relatedness (*r*). The negative correlation between diversity indices (*H* and *I*) and relatedness indicates that inbreeding and genetic drift play a significant role in reducing genetic variability in the studied population which results in increased differentiation among sub-populations. Similar phenomenon was also found in *Arapaima gigas* species [49].

The success of any breeding program usually depends on the right choice of parental groups at the inception. The NDSU flax breeding program is comparatively old. The program already has developed some high yielding and high oil content varieties as well as considerable amount of advanced breeding lines. To enrich the parental stock of the on-going program, the genetic diversity of 350 flax germplasms comprising NDSU released varieties and advanced breeding lines were analyzed in this study. We partitioned the whole collection to its maximum i.e. seven sub-populations based on structure, PCoA and NJ-tree analysis though cluster number was less [43, 50, 51] and more [52] than ours finding in previous studies. The genetic structure among populations is influenced by gene flow, mutation, selection, and mating strategy [53]. In the studied collection of 350 lines, we identified limited gene flow as one of the determinants of genetic differentiation as *Nm* value was less than one [54]. It was also supported by the relatively large separation of P6 (Indian and Pakistani genotypes) and P7 (Turkish genotypes) from other sub-populations as extensive geographic distance hinders the gene flow. Limited gene flow also led to high genetic differentiation in *Calotropis procera* [55], in *Nelumbo lutea* [56] and flax [45]. Sub-population P1, P2 and P3 contained European genotypes where P1 is dominated by Hungarian genotypes, but P2 and P3 contained Hungarian as well as genotypes from other European countries which supports the hypothesis of active exchange of flax germplasms among European countries [57]. The presence of fiber type genotypes in P2 is likely one of the reasons for separation of P2 with other European groups P1 and P3. The P4 is composed of genotypes from closely located African and Asian countries which indicates exchange of genetic material among those countries. As per our expectation, all NDSU released varieties and advanced breeding lines, Canadian genotypes were grouped under the same sub-population (P5) as advanced breeding lines shared ancestors and historical germplasm exchanged occurred between USA and Canada [16]. The results of the mantel test indicated non-significant correlation between genetic and geographic distances of the studied populations. This supports the sporadic presence of genotypes of different origins in same sub-population, especially in P1, P2, P3, P6, P7. A similar scenario also occurred in a previous diversity analysis study of flax due to weak passport data [22]. However, this was not true for P4 and P5 as the mantel test showed significant correlation between geographical distances and the genetic distances. The significant associations between genetic distances and geographical distances were also detected in pale flax and flax collections [58] and in *Linum austriacum* (Lineaceae) populations [59].

Hybridization among genotypes from divergent populations will usually produce more diversity, transgressive segregation, and heterosis resulting in higher genetic gain. Pairwise *F*_*st*_ is a good indication of the degree of divergence among populations. Both high and low pairwise *F*_*st*_ value is good for parent selection depending on the objectives. In the present study, we identified statistically significant large and small pairwise *F*_*st*_ values. Similar results were also found in previous studies [52, 60]. To develop high yielding and high oil content varieties we will choose breeding parents from divergent sub-population pairs such as P5 and P6, P7 and P6 as pairwise *F*_*st*_ between them is highest (*F*_*st*_ > 0.50) These sub-populations also contain different released varieties. For creating dual purpose transgressive segregants, we will choose parents from pair P2 and P6 (*F*_*st*_ > 0.50) as P2 contained mainly fiber type and P6 contained oil type genotypes. For quick fixation of both fiber and oil contributing alleles in single individuals, crosses between genotypes of P2 and P5 will be more effective as pairwise *F*_*st*_ < 0.20. Within sub-populations, crossing among genotypes will also be useful as AMOVA reveals variance among individuals within sub-population covered a larger portion of total variation than variance among sub-population. This result is in line with the previous findings [41, 45, 58, 61], but reverse results were also found in recent studies [62, 63]. In this case, we could utilize P3, P4 and P1 showing high diversity (*h* > 2.30). All sub-populations contained both pure (non-hybrid) as well as admixed genotypes. For parent selection, the pure genotypes will be prioritized. However population diversity tends to inflate the real differentiation between any two pair of individuals as it exploits the alleles that not necessarily come from the same parent or ancestor. IBS coefficients are good to decide what individuals will be crossed to combine positive alleles that historically never have been combined. The molecular kinship or coancestry in self-pollinated crops tends to be higher than that in cross-pollinated crops as heterozygosity reduces the probability of two alleles at a locus of being identical by state [64]. In our study, most of the genotypes had weak relatedness as approximately 80% of pairwise coancestry ranged from 1.12 to 1.50. For identifying specific cross combinations within and among sub-populations, genotypes having low IBS coefficients among them will be utilized.

Most of the economically important traits are quantitative in nature. To develop markers for quantitative traits, association mapping (AM) is used and knowledge of linkage disequilibrium (LD) is useful to determine the number and density of markers and experimental design needed to perform the analysis. Although low LD requires more markers for high resolution, it increases the predictive power of each one [24]. We found that the overall LD of the entire collection was 0.03 and LD decay was not observed within short distance for the entire collection as well as each sub-population. This is because of the autogamous (self-pollination) mating mode of flax [65] and LD declines more slowly in self-pollinated crops where recombination is less effective than in cross-pollinating species [24, 66]. The higher LD level was also found in flax [35] and sesame [67] because of self-pollination. We found the slowest LD decay in P6 as the level of genetic variation captured by the target population influences the extent of LD and LD decay is rapid in landraces and accessions compared to related cultivars [68]. We also analyzed chromosome-wise LD decay to select chromosome-wide marker numbers for AM. Our analysis showed that LD decay was high in chromosome Lu13 and Lu8 and low in chromosome Lu1 and Lu3 which was more rapid than LD decay rates in previous findings [50]. This is may be due to the difference in genotype sets and marker sets. This finding indicates that we need to consider more marker for chromosome Lu13 and Lu8 than other chromosomes for better resolution during AM. The overall findings reveal that for fine mapping of QTL by AM, higher markers should be used according to the population and chromosome-wide LD decay rate. Again, selection of populations having low pairwise *F*_*st*_ with high but similar level of LD will reduce the number of required individuals and markers for AM analysis. However, population structure and cryptic relatedness also affects AM analysis by increasing the false positive rate [69, 70]. To minimize the false positives, we will use a mixed linear model (MLM) with Q-matrix and kinship matrix as covariates [70, 71].

## Conclusions

In the present study, we used highly informative SNP markers which were developed through GBS analysis. The identified SNPs provide a clear picture of genetic structure, diversity, relatedness and linkage disequilibrium of the studied population which leads to higher precision in parent selection for a need-based future breeding program. These markers will also facilitate QTL mapping, association mapping, to allow us to utilize marker-assisted and genomic-selection breeding tools for multiple traits breeding.

## Methods

### Plant materials

**Fig. 6:**
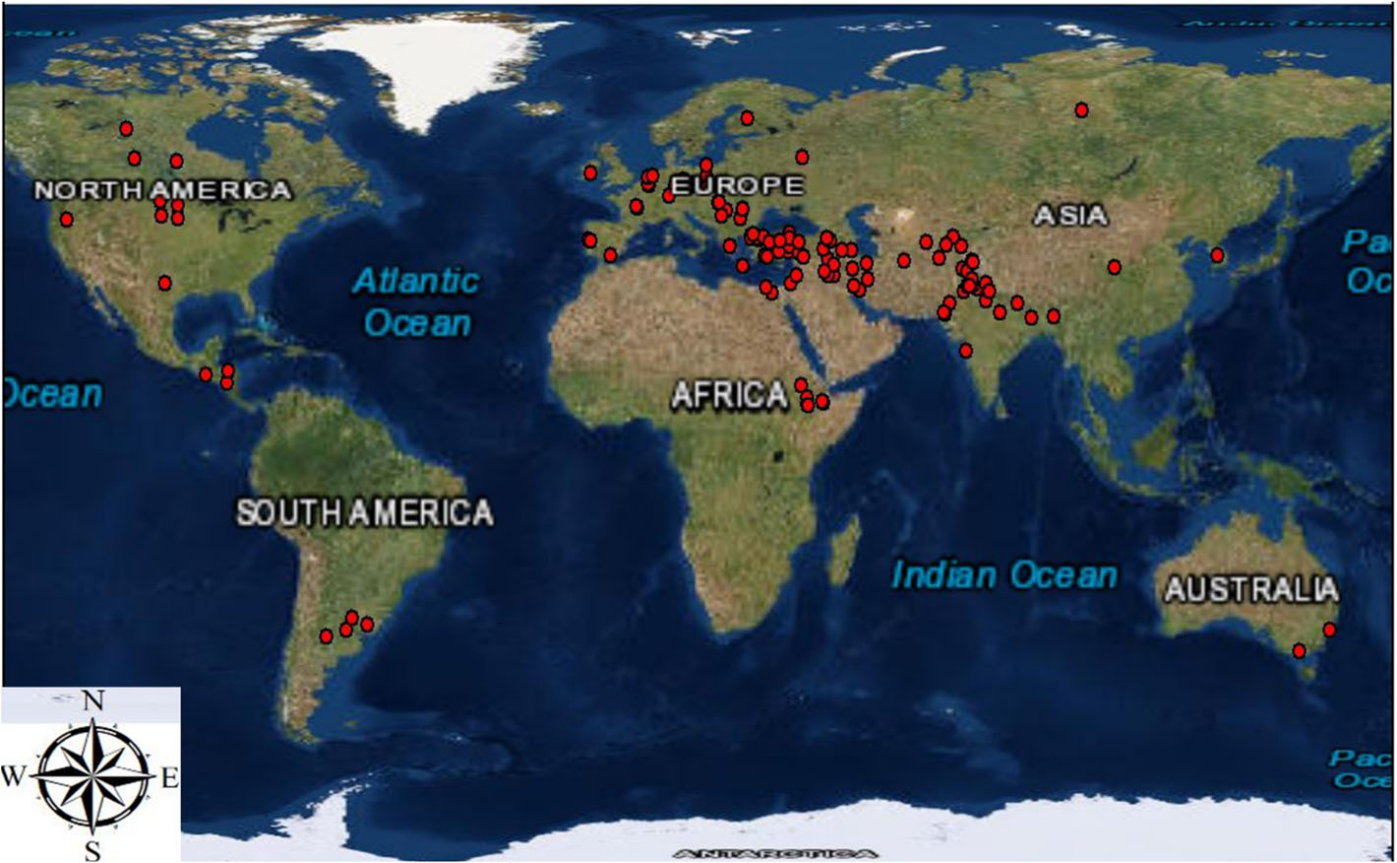
Distribution of genotypes (The map was created using software ArcGIS10.5 trial version, https://www.arcgis.com/features/login/activation.html?activationcode=7c5ef1a364d6625b9482, Subscription ID: 3146087987, accessed on June 25, 2019)

A core collection of 350 flax germplasm accessions originated in 38 countries of 6 continents were collected from North Central Regional Plant Introduction Station (NCRPIS), Ames, Iowa, USA, North Dakota State University (NDSU) released varieties and advanced breeding lines, varieties developed by different institute of USA and Canada (Fig. 6, Table S1).

### DNA extraction and sequencing

Young leaves were collected from 30 days old plants and flash-frozen in liquid nitrogen. Tubes were stored at -80̊ C until lyophilized. The lyophilized leaf tissue was ground in tubes with stainless beads using a plate shaker. DNA was extracted using Qiagen DNeasy Kit (Qiagen, CA, USA) from lyophilized tissue following the manufacturer’s protocol. DNA concentration was measured using a NanoDrop 2000/2000c Spectrophotometer (Thermofisher Scientific). The *ApekI* enzyme was used for GBS library preparation [72]. Sequencing of the library was done at the University of Texas Southwestern Medical Center, Dallas, Texas, USA using Illumina HiSeq 2500 sequencer.

### SNP calling

TASSEL 5 GBSv2 pipeline [73] was used for SNP calling using a 120-base kmer length and minimum kmer count of ten. The reads were aligned to the flax reference genome [23] (available at: ftp://ftp.ncbi.nlm.nih.gov/genomes/all/GCA/000/224/295/GCA_000224295.2_ASM22429v2) using Bowtie 2 (version 2.3.0) alignment tool [74]. After passing all the required steps of TASSEL 5 GBSv2 pipeline, 281,368 unfiltered SNPs were identified. As flax is strictly self-pollinating crop, and this material is assumed to be inbred, all the heterozygous loci were first removed. Heterozygous SNPs are most likely are due to artefactual collapse of homologous sites during alignment. Then VCFtools [75] was used to select bi-allelic SNPs considering the criteria: minor allele frequency (MAF) ≥ 0.05, missing values (max-missing) ≤25%, depth (minDP) ≥ 3 and physical distance (thin) ≤ 500. These filtering steps resulted in a total of 6200 SNP markers.

### Data analysis

The collection was divided into genetic groups using STRUCTURE v2.3.4 [69] software. The admixture model, a burnin period of 10,000 and 50,000 Monte Carlo Markov Chain (MCMC) iterations with 10 replications per K (K1-K10), were used as parameters for structure analysis. The optimal number of groups was determined based on DeltaK approach [76] which was performed by Structure Harvester [77]. The individual Q matrix for the optimal K value was generated utilizing membership coefficient matrices of ten replicates from STRUCTURE analysis using CLUMPP [78]. The results of structure analysis was visualized using the Structure Plot v2 software [79]. Principal co-ordinate analysis (PCoA) was conducted based on Nei’s genetic distance by covariance standardized approach in GenAlex v6.5 [80]. An unrooted neighbor-joining (NJ) phylogenetic tree was constructed using MEGAX program with 1000 bootstrap [81].

Analysis of molecular variance (AMOVA) was done to partition the genetic variance among the groups identified by STRUCTURE in Arlequin3.5 [82]. The average pair-wise between sub-population *Fst* and relatedness (*r*) values were calculated using GenAlex v6.5 [80]. GenAlex v6.5 was also used to estimate percentage of polymorphic loci, number of effective alleles, Shannon’s information index, expected heterozygosity and unbiased expected heterozygosity of each marker and sub-population. The SNP distribution plot was developed using R package CMplot (available at: https://github.com/YinLiLin/R-CMplot). The polymorphism information content (PIC) of markers was calculated using software Cervus [83]. Tajima’s D value of each group was calculated using MEGAX software [81]. The level of relatedness (*r*) was correlated with Shannon’s information index (*I*) and diversity (*H*) in R v3.5.2 [84]. We performed a mantel test [85] within each sub-population based on genetic distance and geographic distance in GenAlex v6.5 as each sub-population was composed of genotypes, collected from different locations. The kinship (IBS) matrix was calculated using software Numericware i [86] and kinship heatmap and histogram were developed using R package ComplexHeatmap [87]. Linkage disequilibrium (LD) pattern of whole collection and different sub-populations were analyzed using PopLDdecay [88].

## Supplementary information

**Additional file 1: Table S1.** List of the genotypes analyzed in this study.

**Additional file 2: Table S2.** Marker diversity parameters.

**Additional file 3: Table S3.** Sub-population wise marker diversity parameters.

**Additional file 4: Table S4.** a Percentage of variation explained by the first 3 axes, b Eigen values by axis and sample Eigen vectors.

**Additional file 5: Table S5.** *Fst* values at loci level.

**Additional file 6: Table S6.** Mean LD values according to distance.

**Additional file 7: Table S7.** Chromosomewise LD decay rate (Kb) within each sub-population.

**Additional file 8: Table S8.** Kinship (IBS) matrix.

**Additional file 9: Figure S1.** Mantel test output for whole collection and each sub-populations.

**Additional file 10: Figure S2a-b.** Chromosome-wise LD decay rate considering whole collection.

**Additional file 11: Figure S3.** Chromosome-wise SNP distribution pattern.

**Additional file 12: Figure S4.** Heatmap of kinship matrix.

**Additional file 13: Figure S5.** Histogram of IBS coefficients.

## Data Availability

The datasets used and/or analyzed during the current study are available from the corresponding author on reasonable request.

## Abbreviations

GBS: Genotype by sequencing
SNP: Single nucleotide polymorphism
MAF: Minor allele frequency
PIC: Polymorphism information content
NJ: Neighbor joining
AMOVA: Analysis of molecular variance
Fst: Fixation index
PCoA: Principle coordinate analysis
IBS: Identical by state
LD: Linkage disequilibrium
AM: Association mapping

## References

[1] Allaby RG, Peterson GW, Merriwether DA, Fu Y-B (2005). Evidence of the domestication history of flax (Linum usitatissimum L.) from genetic diversity of the sad2 locus. Theor Appl Genet..

[2] Westcott ND, Muir AD (2003). Flax seed lignan in disease prevention and health promotion. Phytochem Rev..

[3] Przybylski R (2005). Flax oil and high linolenic oils. Bailey’s Ind Oil fat Prod..

[4] Cullis C (2011). Linum. Wild crop relatives: genomic and breeding resources.

[5] Myles S, Peiffer J, Brown PJ, Ersoz ES, Zhang Z, Costich DE (2009). Association mapping: critical considerations shift from genotyping to experimental design. Plant Cell..

[6] Diederichsen A, Raney JP (2006). Seed colour, seed weight and seed oil content in Linum usitatissimum accessions held by plant gene resources of Canada. Plant Breed..

[7] Saeidi G (2012). Genetic variation and heritability for germination, seed vigour and field emergence in brown and yellow-seeded genotypes of flax. Int J Plant Prod..

[8] Tyson H, Fieldes MA, Cheung C, Starobin J (1985). Isozyme relative mobility (R m) changes related to leaf position; apparently smoothR m trends and some implications. Biochem Genet..

[9] Månsby E, von Díaz O, Von Bothmer R (2000). Preliminary study of genetic diversity in Swedish flax (Linum usitatissimum). Genet Resour Crop Evol..

[10] Van Beuningen LT, Busch RH (1997). Genetic diversity among north American spring wheat cultivars: III. Cluster analysis based on quantitative morphological traits. Crop Sci..

[11] Kuhns LJ, Fretz TA (1978). Distinguishing rose cultivars by polyacrylamide gel electrophoresis. I. Extraction and storage of protein and active enzymes from rose leaves [chemotaxonomy]. J Am Soc Hort Sci..

[12] Falkenhagen ER (1985). Isozyme studies in provenance research of forest trees. Theor Appl Genet..

[13] Eckert RT, Joly RJ, Neale DB (1981). Genetics of isozyme variants and linkage relationships among allozyme loci in 35 eastern white pine clones. Can J For Res..

[14] Tobolski JJ, Kemery RD (1992). Identification of red maple cultivars by isozyme analysis. HortScience..

[15] Bohn M, Utz HF, Melchinger AE (1999). Genetic similarities among winter wheat cultivars determined on the basis of RFLPs, AFLPs, and SSRs and their use for predicting progeny variance. Crop Sci..

[16] Fu Y-B, Rowland GG, Duguid SD, Richards KW (2003). RAPD analysis of 54 north American flax cultivars. Crop Sci..

[17] Everaert I, De Riek J, De Loose M, VAN WAES J, Van Bockstaele E (2001). Most similar variety grouping for distinctness evaluation of flax and linseed (Linum usitatissimum L.) varieties by means of AFLP and morphological data. Plant Var Seeds..

[18] Kumari A, Paul S, Sharma V (2018). Genetic diversity analysis using RAPD and ISSR markers revealed discrete genetic makeup in relation to fibre and oil content in Linum usitatissimum L. genotypes. Nucl..

[19] El Sayed AA, Ezzat SM, Mostafa SH, Zedan SZ, Abdel-Sattar E, El Tanbouly N (2018). Inter simple sequence repeat analysis of genetic diversity and relationship in four egyptian flaxseed genotypes. Pharm Res..

[20] Mhiret WN, Heslop-Harrison JS (2018). Biodiversity in Ethiopian linseed (*Linum usitatissimum* L.): molecular characterization of landraces and some wild species. Genet Resour Crop Evol..

[21] Kumar Yadav H, Chandrawati D, Singh N, Kumar R, Kumar S, Ranade SA (2018). Agro-morphological traits and microsatellite markers based genetic diversity in Indian genotypes of linseed (*Linum usitatissimum* L.). J Agr Sci Tech.

[22] Soto-Cerda BJ, Diederichsen A, Ragupathy R, Cloutier S (2013). Genetic characterization of a core collection of flax (*Linum usitatissimum* L.) suitable for association mapping studies and evidence of divergent selection between fiber and linseed types. BMC Plant Biol.

[23] You FM, Xiao J, Li P, Yao Z, Jia G, He L (2018). Chromosome-scale pseudomolecules refined by optical, physical and genetic maps in flax. Plant J..

[24] Flint-Garcia SA, Thornsberry JM, Buckler ES (2003). Structure of linkage disequilibrium in plants. Annu Rev Plant Biol..

[25] Wright S (1943). An analysis of local variability of flower color in Linanthus parryae. Genetics..

[26] Luo Z, Iaffaldano BJ, Zhuang X, Fresnedo-Ramirez J, Cornish K (2017). Analysis of the first Taraxacum kok-saghyz transcriptome reveals potential rubber yield related SNPs. Sci Rep..

[27] Luo Z, Brock J, Dyer JM, Kutchan TM, Augustin M, Schachtman DP (2019). Genetic diversity and population structure of a Camelina sativa spring panel. Front Plant Sci..

[28] Yang H, Wei C-L, Liu H-W, Wu J-L, Li Z-G, Zhang L (2016). Genetic divergence between Camellia sinensis and its wild relatives revealed via genome-wide SNPs from RAD sequencing. PLoS One..

[29] Mantello CC, Cardoso-Silva CB, da Silva CC, de Souza LM, Junior EJS, de Souza GP (2014). De novo assembly and transcriptome analysis of the rubber tree (Hevea brasiliensis) and SNP markers development for rubber biosynthesis pathways. PLoS One..

[30] Huang S, Deng L, Guan M, Li J, Lu K, Wang H (2013). Identification of genome-wide single nucleotide polymorphisms in allopolyploid crop Brassica napus. BMC Genomics..

[31] Clarke WE, Parkin IA, Gajardo HA, Gerhardt DJ, Higgins E, Sidebottom C (2013). Genomic DNA enrichment using sequence capture microarrays: a novel approach to discover sequence nucleotide polymorphisms (SNP) in Brassica napus L. PLoS One..

[32] Guo C, McDowell IC, Nodzenski M, Scholtens DM, Allen AS, Lowe WL (2017). Transversions have larger regulatory effects than transitions. BMC Genomics..

[33] Shete S, Tiwari H, Elston RC (2000). On estimating the heterozygosity and polymorphism information content value. Theor Popul Biol..

[34] Botstein D, White RL, Skolnick M, Davis RW (1980). Construction of a genetic linkage map in man using restriction fragment length polymorphisms. Am J Hum Genet..

[35] Singh N, Agarwal N, Yadav HK (2019). Genome-wide SNP-based diversity analysis and association mapping in linseed (*Linum usitatissimum* L.). Euphytica.

[36] Eltaher S, Sallam A, Belamkar V, Emara HA, Nower AA, Salem KFM (2018). Genetic diversity and population structure of F3: 6 Nebraska winter wheat genotypes using genotyping-by-sequencing. Front Genet..

[37] Alipour H, Bihamta MR, Mohammadi V, Peyghambari SA, Bai G, Zhang G (2017). Genotyping-by-sequencing (GBS) revealed molecular genetic diversity of Iranian wheat landraces and cultivars. Front Plant Sci..

[38] Ab Razak S, Azman NHEN, Kamaruzaman R, Saidon SA, MFM Y, Ismail SN (2019). Genetic diversity of released Malaysian rice varieties based on single nucleotide polymorphism markers. Czech J Genet Plant Breed..

[39] Ajala SO, Olayiwola MO, Ilesanmi OJ, Gedil M, Job AO, Olaniyan AB (2019). Assessment of genetic diversity among low-nitrogen-tolerant early generation maize inbred lines using SNP markers. South African J Plant Soil..

[40] Coates BS, Sumerford DV, Miller NJ, Kim KS, Sappington TW, Siegfried BD (2009). Comparative performance of single nucleotide polymorphism and microsatellite markers for population genetic analysis. J Hered..

[41] Smýkal P, Bačová-Kerteszová N, Kalendar R, Corander J, Schulman AH, Pavelek M (2011). Genetic diversity of cultivated flax (Linum usitatissimum L.) germplasm assessed by retrotransposon-based markers. Theor Appl Genet..

[42] Chandrawati SN, Kumar R, Kumar S, Singh PK, Yadav VK (2017). Genetic diversity, population structure and association analysis in linseed (*Linum usitatissimum* L.). Physiol Mol Biol Plants..

[43] Habibollahi H, Noormohammadi Z, Sheidai M, Farahani F (2016). SSR and EST-SSR-based population genetic structure of Linum L.(Linaceae) species in Iran. Genet Resour Crop Evol..

[44] Choudhary SB, Sharma HK, Kumar AA, Maruthi RT, Mitra J, Chowdhury I (2017). SSR and morphological trait based population structure analysis of 130 diverse flax (Linum usitatissimum L.) accessions. C R Biol..

[45] Soto-Cerda BJ, Maureira-Butler I, Muñoz G, Rupayan A, Cloutier S (2012). SSR-based population structure, molecular diversity and linkage disequilibrium analysis of a collection of flax (Linum usitatissimum L.) varying for mucilage seed-coat content. Mol Breed..

[46] Monfared MA, Samsampour D, Sharifi-Sirchi GR, Sadeghi F (2018). Assessment of genetic diversity in Salvadora persica L. based on inter simple sequence repeat (ISSR) genetic marker. J Genet Eng Biotechnol..

[47] Tajima F (1989). Statistical method for testing the neutral mutation hypothesis by DNA polymorphism. Genetics..

[48] Fu Y-B, Diederichsen A, Allaby RG (2012). Locus-specific view of flax domestication history. Ecol Evol..

[49] Torati LS, Taggart JB, Varela ES, Araripe J, Wehner S, Migaud H (2019). Genetic diversity and structure in Arapaima gigas populations from Amazon and Araguaia-Tocantins river basins. BMC Genet..

[50] Soto-Cerda B, Cloutier S, Quian R, Gajardo H, Olivos M, You F (2018). Genome-wide association analysis of mucilage and hull content in flax (*Linum usitatissimum* L.) seeds. Int J Mol Sci.

[51] Saha D, Rana RS, Das S, Datta S, Mitra J, Cloutier SJ (2019). Genome-wide regulatory gene-derived SSRs reveal genetic differentiation and population structure in fiber flax genotypes. J Appl Genet..

[52] Sertse D, You FM, Ravichandran S, Cloutier S (2019). The genetic structure of flax illustrates environmental and anthropogenic selections that gave rise to its eco-geographical adaptation. Mol Phylogenet Evol..

[53] Schaal BA, Hayworth DA, Olsen KM, Rauscher JT, Smith WA (1998). Phylogeographic studies in plants: problems and prospects. Mol Ecol..

[54] Wright S (1965). The interpretation of population structure by F-statistics with special regard to systems of mating. Evolution (N Y)..

[55] Muriira NG, Muchugi A, Yu A, Xu J, Liu A (2018). Genetic diversity analysis reveals genetic differentiation and strong population structure in calotropis plants. Sci Rep..

[56] Islam MR, Zhang Y, Li Z-Z, Liu H, Chen J-M, Yang X-Y (2020). Genetic diversity, population structure, and historical gene flow of Nelumbo lutea in USA using microsatellite markers. Aquat Bot..

[57] Maggioni L (2002). Flax genetic resources in Europe: ad hoc meeting, 7–8 December 2001.

[58] Uysal H, Fu Y-B, Kurt O, Peterson GW, Diederichsen A, Kusters P (2010). Genetic diversity of cultivated flax (Linum usitatissimum L.) and its wild progenitor pale flax (Linum bienne mill.) as revealed by ISSR markers. Genet Resour Crop Evol..

[59] Sheidai M, Afshar F, Keshavarzi M, Talebi S-M, Noormohammadi Z, Shafaf T (2014). Genetic diversity and genome size variability in Linum austriacum (Lineaceae) populations. Biochem Syst Ecol..

[60] Fu Y-B (2012). Population-based resequencing revealed an ancestral winter group of cultivated flax: implication for flax domestication processes. Ecol Evol..

[61] Soto-Cerda BJ, Diederichsen A, Duguid S, Booker H, Rowland G, Cloutier S (2014). The potential of pale flax as a source of useful genetic variation for cultivated flax revealed through molecular diversity and association analyses. Mol Breed..

[62] Habibollahi H, Noormohammadi Z, Sheidai M, Farahani F (2015). Genetic structure of cultivated flax (Linum usitatissimum L.) based on retrotransposon-based markers. Genetika..

[63] Noormohammadi Z, Shafaf T, Farahani F, Sheidai M, Talebi SM, Farahani YH-A (2015). Within and among-genetic variation in Asian flax *Linum austriacum* (Linaceae) in response to latitude changes: cytogenetic and molecular analyses. Biodiversitas J Biol Divers..

[64] Bernardo R, Romero-Severson J, Ziegle J, Hauser J, Joe L, Hookstra G (2000). Parental contribution and coefficient of coancestry among maize inbreds: pedigree, RFLP, and SSR data. Theor Appl Genet..

[65] Vos PG, Paulo MJ, Voorrips RE, Visser RGF, van Eck HJ, van Eeuwijk FA (2017). Evaluation of LD decay and various LD-decay estimators in simulated and SNP-array data of tetraploid potato. Theor Appl Genet..

[66] Abdurakhmonov IY, Abdukarimov A (2008). Application of association mapping to understanding the genetic diversity of plant germplasm resources. Int J Plant Genomics.

[67] Cui C, Mei H, Liu Y, Zhang H, Zheng Y (2017). Genetic diversity, population structure, and linkage disequilibrium of an association-mapping panel revealed by genome-wide SNP markers in sesame. Front Plant Sci..

[68] Xu J, Ranc N, Muños S, Rolland S, Bouchet J-P, Desplat N (2013). Phenotypic diversity and association mapping for fruit quality traits in cultivated tomato and related species. Theor Appl Genet..

[69] Pritchard JK, Stephens M, Donnelly P (2000). Inference of population structure using multilocus genotype data. Genetics..

[70] Yu J, Pressoir G, Briggs WH, Bi IV, Yamasaki M, Doebley JF (2006). A unified mixed-model method for association mapping that accounts for multiple levels of relatedness. Nat Genet..

[71] Price AL, Patterson NJ, Plenge RM, Weinblatt ME, Shadick NA, Reich D (2006). Principal components analysis corrects for stratification in genome-wide association studies. Nat Genet..

[72] Elshire RJ, Glaubitz JC, Sun Q, Poland JA, Kawamoto K, Buckler ES (2011). A robust, simple genotyping-by-sequencing (GBS) approach for high diversity species. PLoS One..

[73] Glaubitz JC, Casstevens TM, Lu F, Harriman J, Elshire RJ, Sun Q (2014). TASSEL-GBS: a high capacity genotyping by sequencing analysis pipeline. PLoS One..

[74] Langmead B, Salzberg SL (2012). Fast gapped-read alignment with bowtie 2. Nat Methods..

[75] Danecek P, Auton A, Abecasis G, Albers CA, Banks E, DePristo MA (2011). The variant call format and VCFtools. Bioinformatics..

[76] Evanno G, Regnaut S, Goudet J (2005). Detecting the number of clusters of individuals using the software STRUCTURE: a simulation study. Mol Ecol..

[77] Earl DA (2012). Others. STRUCTURE HARVESTER: a website and program for visualizing STRUCTURE output and implementing the Evanno method. Conserv Genet Resour..

[78] Jakobsson M, Rosenberg NA (2007). CLUMPP: a cluster matching and permutation program for dealing with label switching and multimodality in analysis of population structure. Bioinformatics..

[79] Ramasamy RK, Ramasamy S, Bindroo BB, Naik VG (2014). STRUCTURE PLOT: a program for drawing elegant STRUCTURE bar plots in user friendly interface. Springerplus..

[80] Peakall R, Smouse PE (2012). GenAlEx 6.5: genetic analysis in excel. Population genetic software for teaching and research—an update. Bioinformatics..

[81] Kumar S, Stecher G, Li M, Knyaz C, Tamura K (2018). MEGA X: molecular evolutionary genetics analysis across computing platforms. Mol Biol Evol..

[82] Excoffier L, Lischer HEL (2010). Arlequin suite ver 3.5: a new series of programs to perform population genetics analyses under Linux and windows. Mol Ecol Resour..

[83] Slate J, Marshall T, Pemberton J (2000). A retrospective assessment of the accuracy of the paternity inference program CERVUS. Mol Ecol..

[84] R Core Team. R: a language and environment for statistical computing. Vienna; 2019. Available from: https://www.r-project.org/.

[85] Podani J (2000). Introduction to the exploration of multivariate data [English translation].

[86] Kim B, Beavis WD (2017). Numericware i: identical by state matrix calculator. Evol Bioinforma..

[87] Gu Z, Eils R, Schlesner M (2016). Complex heatmaps reveal patterns and correlations in multidimensional genomic data. Bioinformatics..

[88] Zhang C, Dong S-S, Xu J-Y, He W-M, Yang T-L (2018). PopLDdecay: a fast and effective tool for linkage disequilibrium decay analysis based on variant call format files. Bioinformatics..

