# Supplementary figures and images for: Genetic diversity analysis of a flax (Linum usitatissimum L.) global collection
Source: BMC Genomics. 2020 Aug 14;21:557. doi: 10.1186/s12864-020-06922-2 (PMC7430851; doi:10.1186/s12864-020-06922-2)

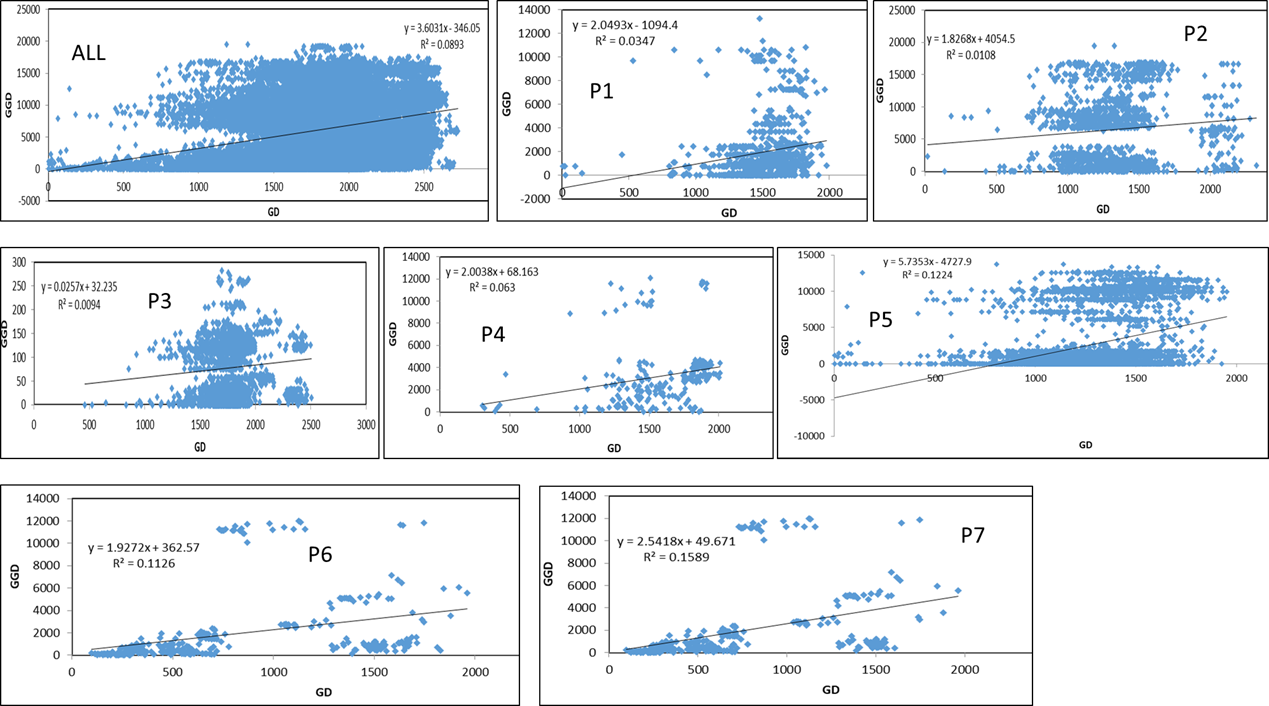

Supplement: Supplementary file 9 — Additional file 9: Figure S1. Mantel test output for whole collection and each sub-populations. [file 12864_2020_6922_MOESM9_ESM.tif]

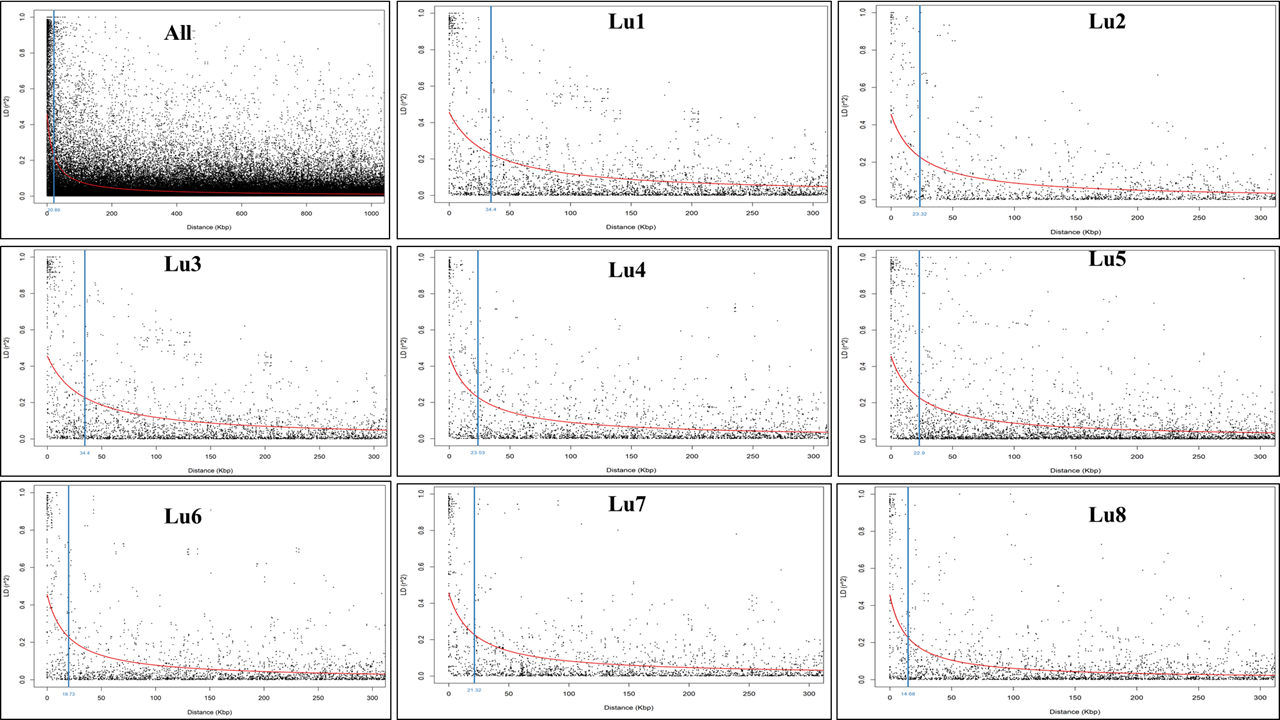

Supplement: Supplementary file 10 — Additional file 10: Figure S2a-b. Chromosome-wise LD decay rate considering whole collection. [file 12864_2020_6922_MOESM10_ESM.zip › Additional file 10 Figure S2a.tif]

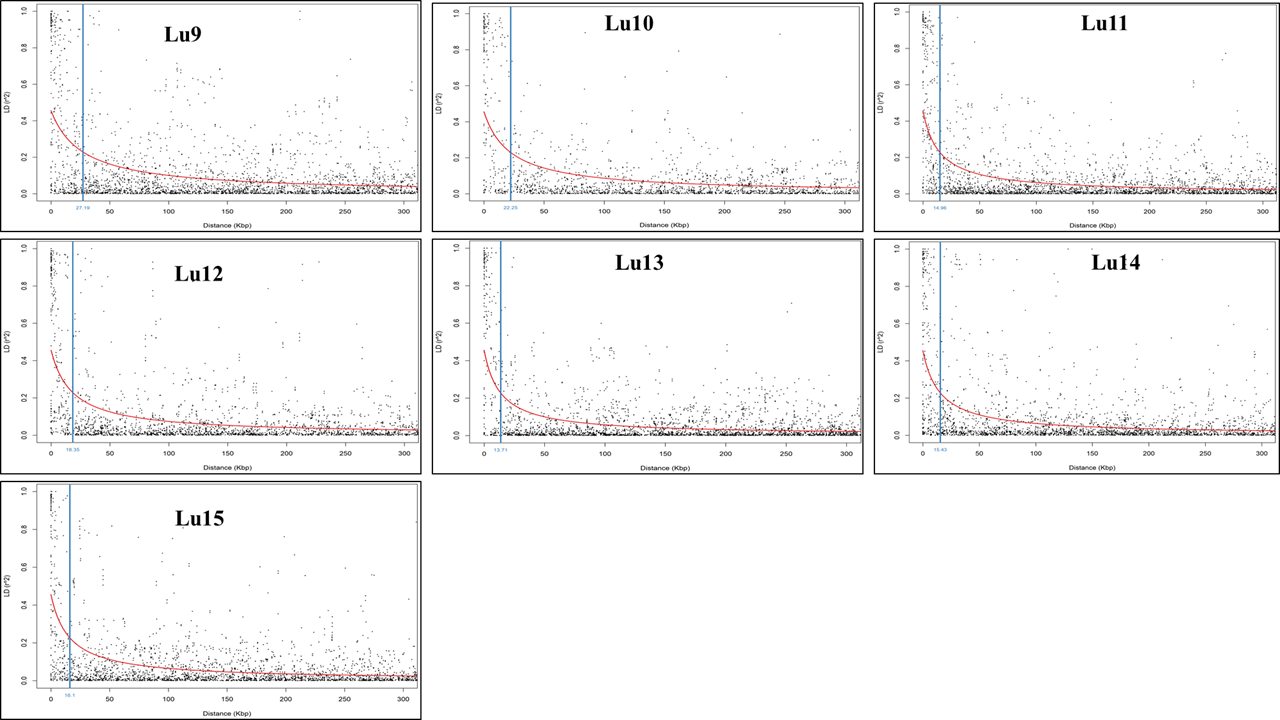

Supplement: Supplementary file 10 — Additional file 10: Figure S2a-b. Chromosome-wise LD decay rate considering whole collection. [file 12864_2020_6922_MOESM10_ESM.zip › Additional file 10 Figure S2b.tif]

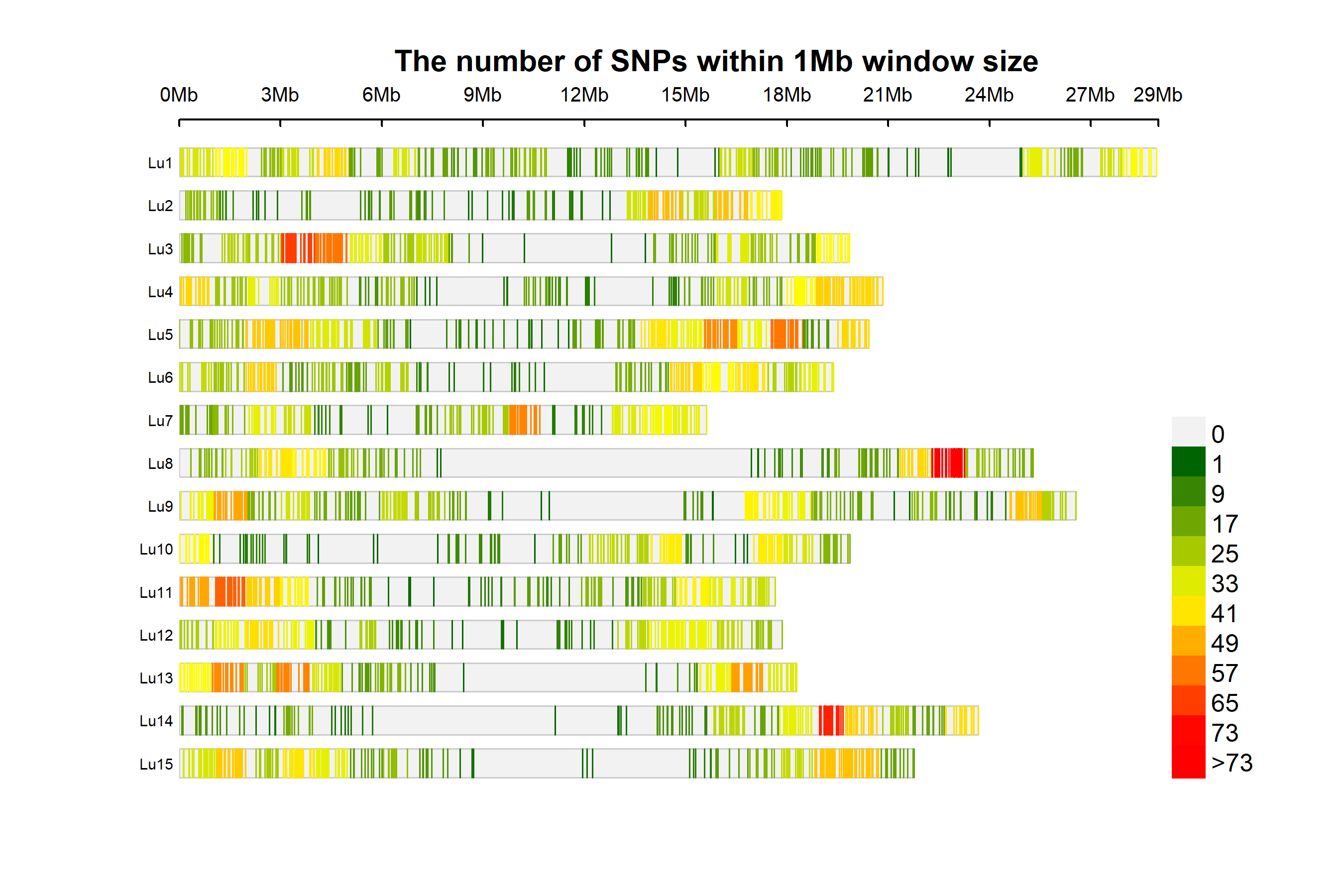

Supplement: Supplementary file 11 — Additional file 11: Figure S3. Chromosome-wise SNP distribution pattern. [file 12864_2020_6922_MOESM11_ESM.tiff]

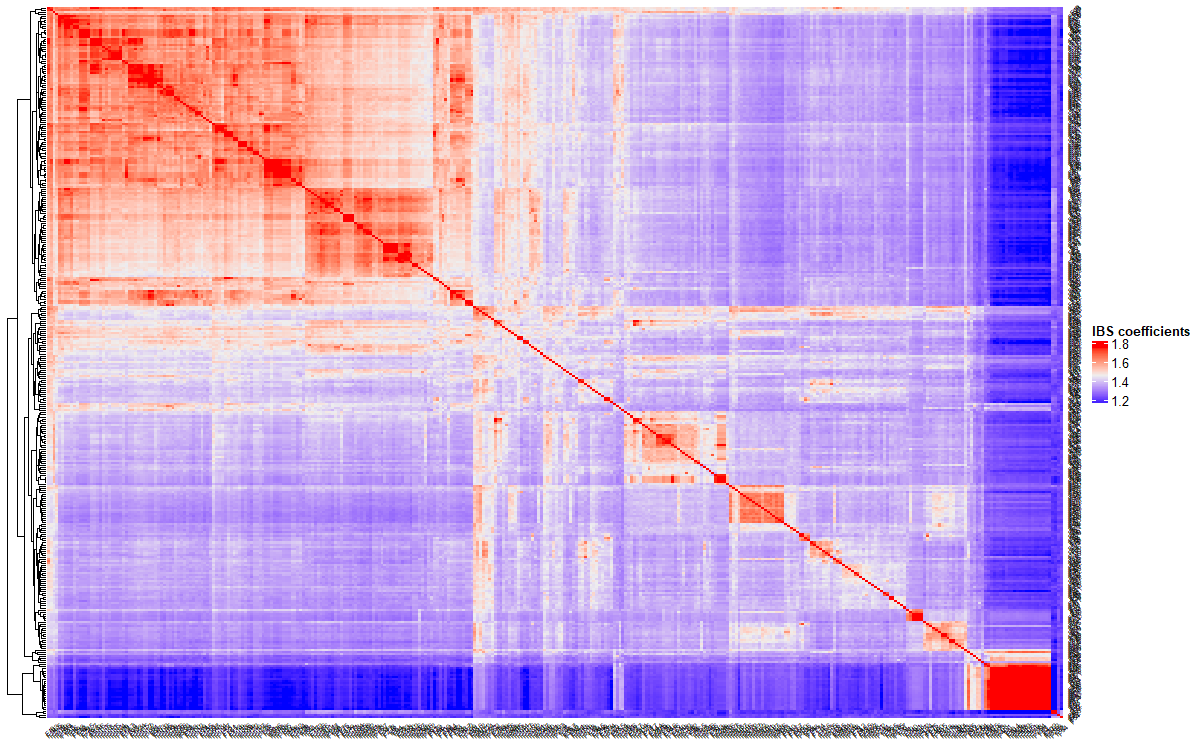

Supplement: Supplementary file 12 — Additional file 12: Figure S4. Heatmap of kinship matrix. [file 12864_2020_6922_MOESM12_ESM.tiff]

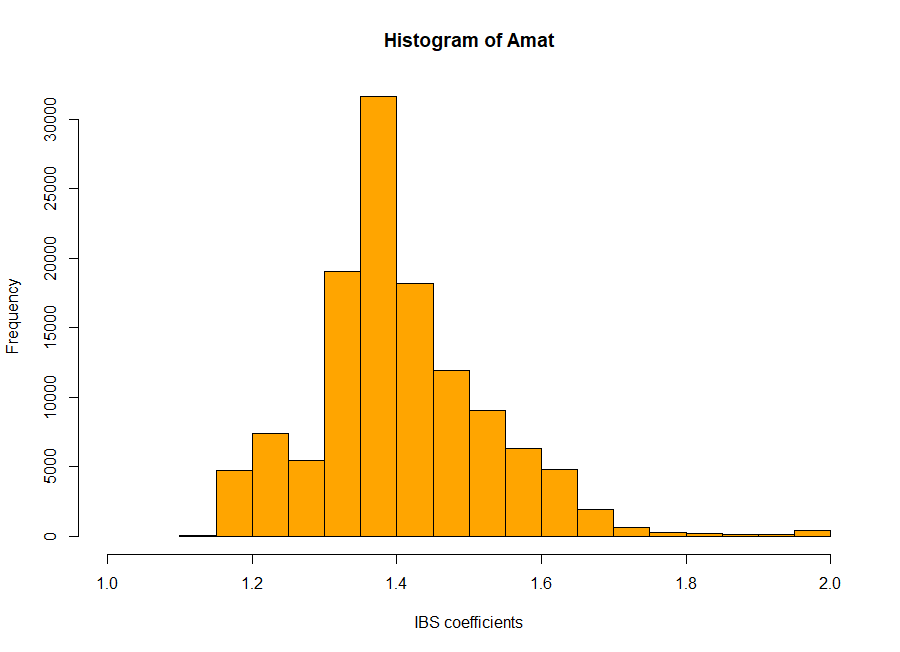

Supplement: Supplementary file 13 — Additional file 13: Figure S5. Histogram of IBS coefficients. [file 12864_2020_6922_MOESM13_ESM.tiff]
